# Lasting organ-level bone mechanoadaptation is unrelated to local strain

**DOI:** 10.1126/sciadv.aax8301

**Published:** 2020-03-06

**Authors:** Behzad Javaheri, Hajar Razi, Stephanie Gohin, Sebastian Wylie, Yu-Mei Chang, Phil Salmon, Peter D. Lee, Andrew A. Pitsillides

**Affiliations:** 1 Skeletal Biology Group, Comparative Biomedical Sciences, The Royal Veterinary College, Royal College Street, London NW1 0TU, UK.; 2 Max Planck Institute of Colloids and Interfaces, Department of Biomaterials, Research Campus Golm, 14424 Potsdam, Germany.; 3 Cluster of Excellence, Humboldt University of Berlin, Berlin, Germany.; 4 Bruker microCT, Kartuizersweg 3B, 2550 Kontich, Belgium.; 5 Mechanical Engineering, University College London, London WC1E 7JE, UK.

## Abstract

Bones adapt to mechanical forces according to strict principles predicting straight shape. Most bones are, however, paradoxically curved. To solve this paradox, we used computed tomography–based, four-dimensional imaging methods and computational analysis to monitor acute and chronic whole-bone shape adaptation and remodeling in vivo. We first confirmed that some acute load-induced structural changes are reversible, adhere to the linear strain magnitude regulation of remodeling activities, and are restricted to bone regions in which marked antiresorptive actions are evident. We make the novel observation that loading exerts significant lasting modifications in tibial shape and mass across extensive bone regions, underpinned by (re)modeling independent of local strain magnitude, occurring at sites where the initial response to load is principally osteogenic. This is the first report to demonstrate that bone loading stimulates nonlinear remodeling responses to strain that culminate in greater curvature adjusted for load predictability without sacrificing strength.

## Teaser

Bones’ four-dimensional response to momentary loading is lasting and coordinated at the organ level.

## INTRODUCTION

It is proposed that bone is protected against failure by a mechanoadaptive process via which skeletal mass and architecture are modified in response to the load environment (*1*). This idea is not new. Galileo Galilei speculated that the shape of bones is related to loading, and Julius Wolff later theorized that “every change in the form and function of bones, or of their function alone, is followed by certain definite changes in their internal architecture and equally definite secondary alteration in their external conformation in accordance with mechanical laws” (*2*). Despite these long-held views, the deciphering of the mechanoadaptive mechanisms that act to secure organ-level protection against load-related failure is only fragmentary.

Engineering principles dictate that curved bones are more prone to failure than straight bones (*3*) and that the mechanoadaptive response to an increase in applied load will, therefore, promote bone straightening. This ideal straight design conflicts with the form of almost all long bones that are, however, curved. Many studies have attempted to deconstruct this contradiction in design by assessment of deformation in curved bones using strain gauges or finite element modeling (FEM) (*4*) under load (*5*), while others have examined the links between body mass and bone curvature (*6*). It has been shown that removing load from rat tibiae by neurectomy and tenotomy results in bones with less than normal curvature. Because these studies did not, however, clarify whether the bone did not develop its normal curvature or was initially curved and became straighter, it is apparent that the enduring effects of increased loading on bone shape remain somewhat speculative.

Bone shape is governed by growth-related endochondral ossification and (re)modeling drift after birth. The latter is considered to be guided primarily by mechanoadaptive regulation of bone quantity (mass), quality (mineralization), and shape [three-dimensional (3D) distribution]. These characteristics are tightly controlled so that modest increases in mass at specific locations result in large biomechanical benefits. Many questions regarding how loading influences bone shape are unanswered. Is there a spatial hierarchy in the changes which loading evokes? Do any load-induced shape changes persist to modify baseline architecture? Which (re)modeling processes dominate the different temporal phases of the mechanoadaptive response, and do these correlate with tissue-level strains?

This last question arises largely due to a theoretical framework proposed by Frost (*7*) that offers a mechanical underpinning for all bone architectures. He hypothesized that strain magnitude serves as the regulatory stimulus for mechanoadaptation, and this was subject to a strain-controlled feedback loop, which was coined the “mechanostat” (*7*). This mechanostat concept predicts that bone is resorbed below a certain threshold strain level, to remove the excess mass, and accrued above another to increase load-bearing strength. Within this framework, major advances emerged concerning the role of mechanics in bone homeostasis in postnatal growth, maturation, and aging (*8*, *9*). Many studies showed that a range of strain-related characteristics, such as rate, frequency, and rest duration between loading cycles can influence functional bone adaptation (*10*). These studies are, however, somewhat limited as they are confined to a small region of the bone, thus precluding conclusions about how overall bone shape responses might be coordinated.

Another inherent component of this mechanostat is the proposed existence of a “strain-controlled feedback loop” to ensure an efficient return to basal bone architectures upon load stimulus removal. However, the findings from epidemiological studies that examine whether increased load bearing engenders any enduring effects, question the legitimacy of inferring its existence. To our knowledge, the longevity of load-induced architectural bone changes has not previously been examined in an experimental model, and there is still controversy regarding the time frame over which the beneficial effects of loading persist.

Experimental studies from in vivo loading models indicate that formation occurs at sites of highest strain, and resorption where strains are lowest (*11*, *12*). This spatial uncoupling in remodeling activities is thought to allow for mechanoadaptive modifications in the shape of the bone, as an organ, in response to applied load (*13*). It is evident, however, that most studies have interrogated responses at specific bone sites rather than along an entire organ’s length (*14*), assuming that the response examined always contributes to strain magnitude–dependent modifications in bone shape. There are no experimental studies to date which have defined the location and extent of load-related resorption and formation at the whole-bone level, nor have any explored (re)modeling activities during the postload period to characterize whether load-related (re)modeling responses are simply reversed with a return to habitual use. Longitudinal microcomputed tomography (microCT) in vivo scanning together with algorithms that permit registration of sequenced images of the same bone at distinct time points, with their respective mechanical stimulation, allows visualization of (re)modeling surfaces across an entire bone’s length, giving the 4D analyses that allow these questions to be addressed.

This study uses a well-established in vivo mouse tibial loading model (*15*), combined with longitudinal imaging by in vivo microCT at multiple time points before and after loading, to precisely describe bone shape alterations, along with a regional evaluation of load-related (re)modeling of almost the entire bone cortex. Our experiments will test Frost’s mechanostat theory in 4D, with time as a variable, to determine whether the adaptive changes in formation and resorption induced by a defined loading regime are simply reversed with a return to solely habitual use (*16*). Coupling with finite element (FE) analysis of cortical bone surface strains allows for relationships to the local mechanical environment to also be explored. For example, are the magnitudes of applied strain and the adaptive response related spatially in a linear or nonlinear manner? Our studies reveal significant acute changes in bone shape that are retained chronically only in specific tibia regions. In accord with the mechanostat theory, much of the load-induced bone accrual in proximal tibia regions is indeed simply resorbed in a strain-dependent manner upon removal of the original osteogenic load stimulus. In contrast with the mechanostat theory, however, we find chronic preservation of load-induced gains in mid-to-distal tibia regions that are independent of strain magnitude, thus exhibiting nonlinearity. These lasting modifications in bone shape and mass nonetheless contribute to an increased overall curvature, demonstrating that the mechanoadaptive response generates persistent benefits in the bone at the organ level to generate a structural “memory” of the applied loading environment.

## RESULTS

### Loading drives significant acute and chronic changes in tibial shape

**Fig. 1:**
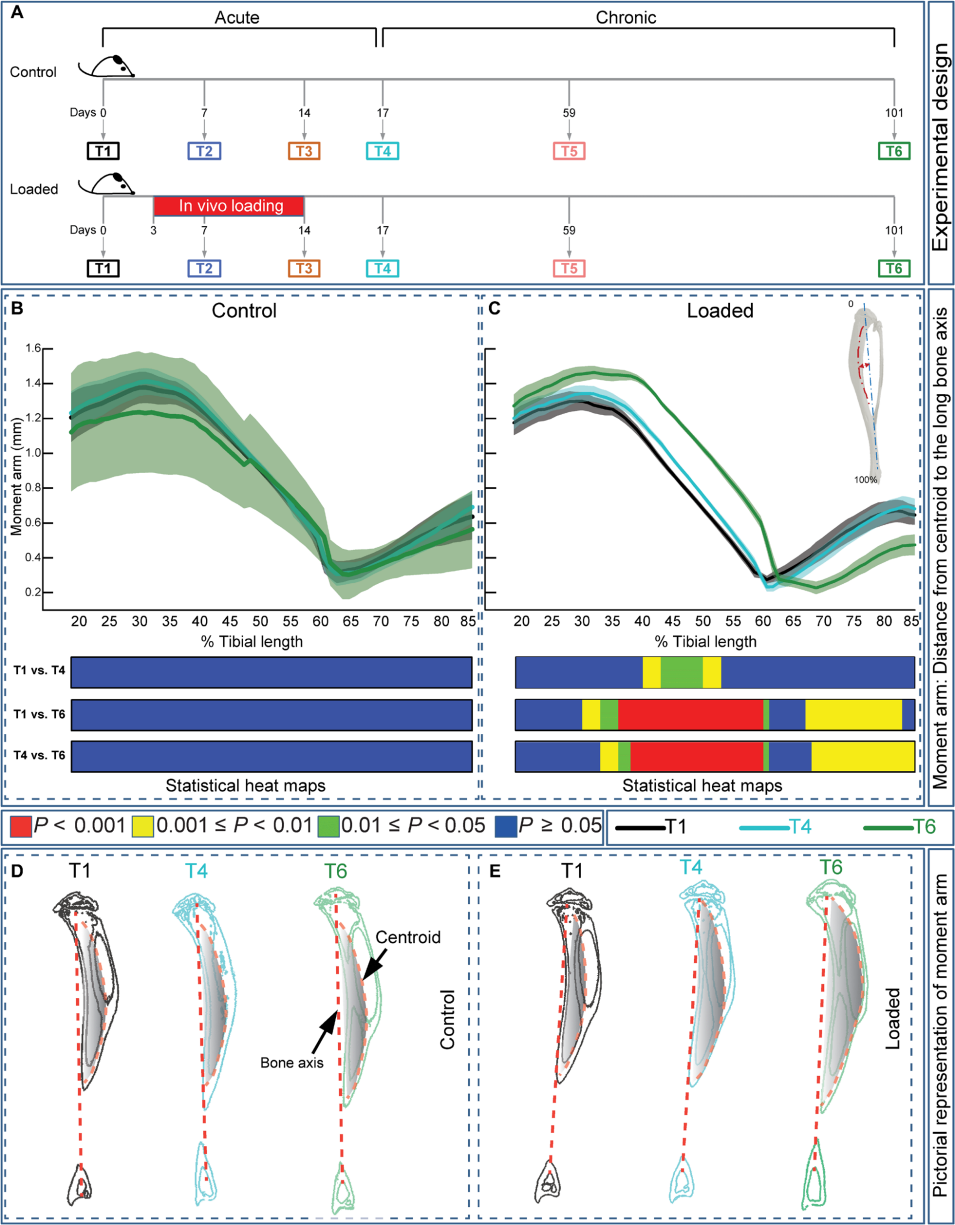
Loading modifies tibial curvature. (**A**) Study design demonstrating the temporal relationship between the sequence of the six in vivo scans (T1–T6) and tibial loading (nonloaded control and loaded mice shown). (**B** and **C**) Tibial curvature along the length in the control and loaded groups. Statistical differences in tibial curvature between different T’s within the control (B) and loaded (C) groups along the entire tibia shaft represented as a heat map. Red, *P* < 0.001; yellow, 0.001 ≤ *P* < 0.01; green, 0.01 ≤ *P* < 0.05; blue, *P* ≥ 0.05. Line graphs represent means ± SEM. (**D** and **E**) Pictorial representation of tibial curvature at T1, T4, and T6 in the control (D) and loaded group (E).

To allow separate evaluation of acute bone changes from those linked with later return to equilibrium mechanical demands, we divided data from longitudinal analyses into two phases. An acute phase that encompasses the initial 17 days (scans T1–T4), ending 3 days after cessation of loading (T4), and a chronic phase from the T4 postload time point to 84 days later (day 101, scans T4–T6; see Fig. 1A). Analysis of tibial curvature during these two phases showed no acute (T1–T4) or chronic (T1–T6 or T4–T6) differences in the control, growth-only group (Fig. 1B). In contrast, loaded limbs exhibited significant acute increases in curvature at ~40 to 55% of the tibia length (Fig. 1C). This load-related effect culminates in the chronic phase, with greater curvature over a sector from ~30 to 60% and a significant reduction over ~70 to 85% of tibia length (Fig. 1C). Our data show that loading leads to acute and further chronic increases in proximal tibia curvature and a reduction at locations distal to the tibiofibular junction (Fig. 1D).

**Fig. 2:**
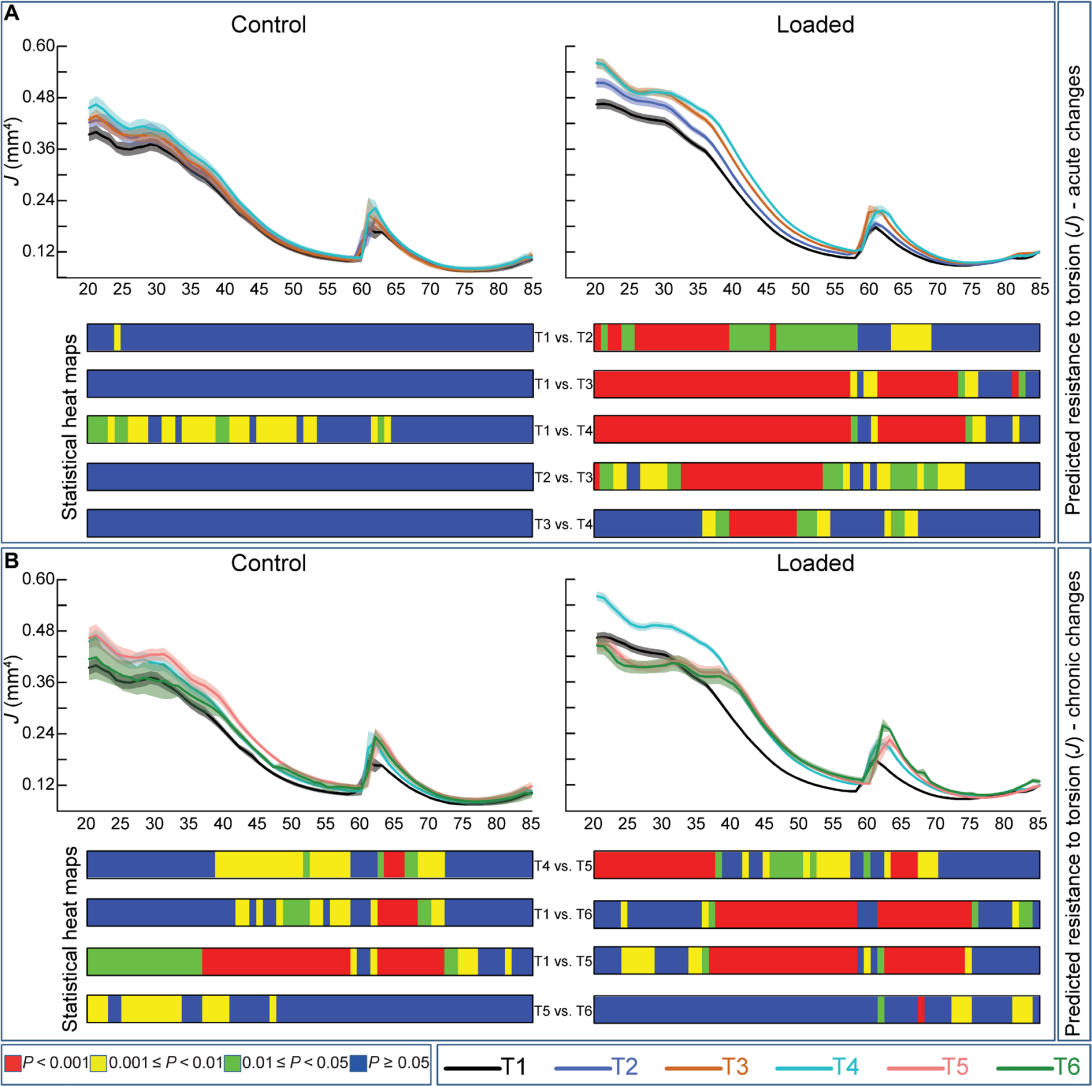
Acute and chronic load-induced changes in predicted resistance to torsion (*J*) along the entire tibia length. (**A**) Acute mean *J* of control and loaded tibiae in female C57/Bl6 at T1–T4. (**B**) Chronic mean *J* of both groups at T1, T4–6 demonstrating chronic mechanoadaptation. Statistical significance of differences in *J* between different T’s within the group along the entire tibia shaft represented as a heat map. Red, *P* < 0.001; yellow, 0.001 ≤ *P* < 0.01; green, 0.01 ≤ *P* < 0.05; blue, *P* ≥ 0.05. Line graphs represent means ± SEM.

Further analyses showed that growth alone in control tibiae is linked only to minor changes in ellipticity (fig. S1). Loading, however, produced significant increases in ellipticity in mid-distal regions (T1–T4, ~40 to 80%) and reduced ellipticity proximally (~25 to 30%; fig. S1A). Significant chronic load-related reshaping of tibia ellipticity was observed in distal and proximal regions, at T4–T5 and T5–T6, respectively (fig. S1B). We also find only minor, acute (T1–T4) enhancement of predicted resistance to torsion (*J*) in control tibiae, while, in contrast, loading leads to very rapid increases in *J* (T1–T2, ~20 to 70%; Fig. 2A), which become more extensive at later time points during the acute phase (T1–T3 and T1–T4). While age-related, chronic phase decline in *J* was observed in the control group, increases in *J* were preserved in the loaded (35 to 75%) but reverted back to original levels on control tibiae during the chronic phase (T1–T5 and T1–T6; Fig. 2B). These data demonstrate lasting tibia shape modifications long after the cessation of mechanoadaptive loading.

### Selective load-induced increases in cortical bone mass are lasting

**Fig. 3:**
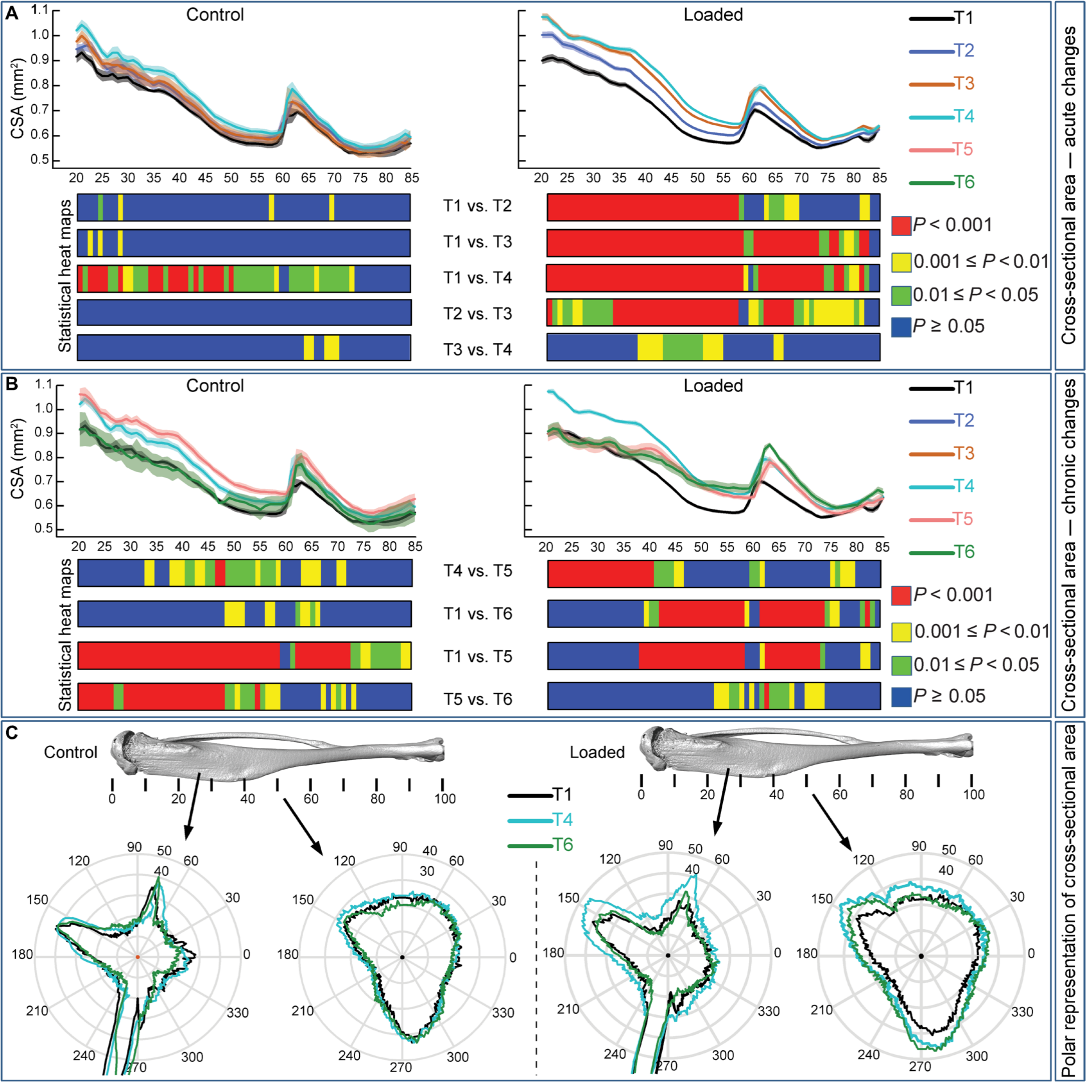
Acute and chronic load-induced adaptation of CSA along the entire tibia length. (**A**) Acute mean CSA of control and loaded tibiae in female C57/Bl6 at T1–T4. (**B**) Chronic mean CSA of both groups at T1, T4–T6 demonstrating chronic mechanoadaptation. (**C**) Acute and chronic polar representation of CSA for the control and loaded groups. Statistical significance of differences in CSA between different T’s within the group along the entire tibia shaft represented as a heat map. Red, *P* < 0.001; yellow, 0.001 ≤ *P* < 0.01; green, 0.01 ≤ *P* < 0.05; blue, *P* ≥ 0.05. Line graphs represent means ± SEM.

Applied load triggered very rapid increases in cortical cross-sectional area (CSA) within the first week (T1–T2) at ~20 to 70%. These continued through the second week of loading, further elevating CSA now also in more distal regions (T2–T3). Load-related increases in CSA were sustained until T4 in a selected 37 to 55% tibial region. In contrast, the analysis found no major acute modification in CSA in control tibiae between T1 and T3, and that accumulated growth raised CSA by T4 (T1–T4) along much of the tibia length (~20 to 75%). These data indicate that the trajectory of load-related bone accrual shows regional differences and that the proximal region, where CSA rises most rapidly, exhibits the least prolonged loading response (Fig. 3A). Analysis of acute (T1–T4) alterations in mean cortical cross-sectional thickness (Cs.Th) also revealed changes closely resembling the patterns seen for CSA (fig. S2A).

### Not all acute load-induced bone mass gains are lost rapidly upon the cessation of the load

In line with ongoing growth, our analyses of CSA between later T4 and T5 time points show that restricted regions (40 to 65%) of control cortices continue to expand and that CSA diminishes after skeletal maturity (T5–T6) along almost the entire tibia length. In contrast, analysis of loaded tibiae during the chronic phase shows that the newly acquired, acute load-related increases in CSA are lost from only a relatively short proximal region (20 to 37%) within the first 6 weeks of the chronic phase (T4–T5 and T1–T5; Fig. 3B). To explore these regional differences further, we evaluated spatial CSA changes at two defined tibia positions, across the acute and chronic phases. Using polar coordinates to map circumferential changes, we found by the end of the study (T6) that control bones showed a complete reversal of the acute growth-related bone gains (T1–T4) at both 25 and 50% positions (Fig. 3C). This reversal was also apparent in the chronic phase at the 25% position in loaded tibiae but was clearly absent at 50%, where load permanently modified cross-sectional bone mass. Comparison of chronic changes in Cs.Th across T1–T6 shows that Cs.Th also remains elevated at 20 to 35% and ~45 to 80% of loaded tibia length (fig. S2B).

These data demonstrate a divergence in the time scale over which mechanoadaptive changes are retained at different regions of a single bone. We find that the acute load-induced responses that appear somewhat later are preferentially retained during an extensive postload phase to yield stable load-specific modifications in cortical mass and shape (versus T1); their chronic character is evidenced by significant postload increases in CSA between T5 and T6 in a clustered distal region (60 to 65%).

### Bone retains regional benefits of load-related (re)modeling

**Fig. 4:**
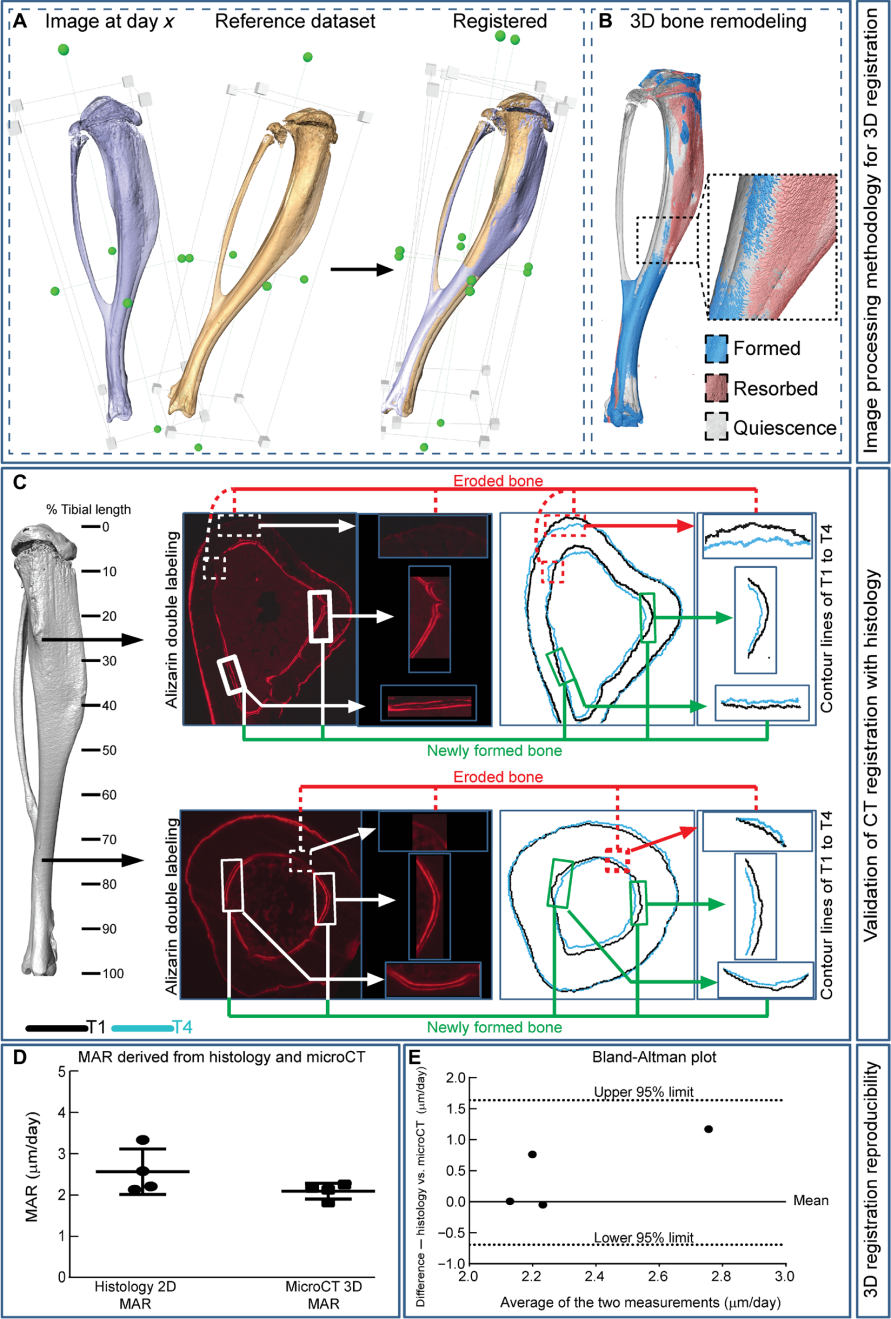
Validation of microCT-based 3D registration. (**A**) Image processing methodology to identify (re)modeling events. Two images obtained at different T’s are superimposed for rigid 3D registration to produce registered datasets. (**B**) These are subsequently used to calculate 3D formation and resorption bone (re)modeling responses. (**C**) Visualization of whole tibia showing location of the region selected for dynamic histomorphometric analysis of alizarin red double labeling (left pair of panels) and corresponding 3D microCT-based morphometry (right pair of panels), showing that areas of newly formed and eroded bone are found in comparable matching regions of the bone cortex. (**D**) MAR (μm/day) determined using 2D histomorphometry and 3D microCT-based morphometry. (**E**) Comparison between MAR assessed by histology and computationally visualized in a Bland-Altman plot.

To assess (re)modeling activities underpinning these load-related bone shape and mass changes, we used the approach of Waarsing *et al.* (*17*). This entails superimposition of repeat 3D scans of the same bone and registration-based measurement of formation and resorption (Fig. 4, A and B) (*17*). This was validated by repeat 3D registration of the same scans and also repeat scans of the same bone, which both showed less than one-voxel (<9 μm) variation, demonstrating high reproducibility. These scan-based methods were further validated against traditional bone histomorphometry (Fig. 4C). Consistent with previous validation, no significant difference in measured mineral apposition rate (MAR) was observed between the two methods (Fig. 4D) (*18*); a Bland–Altman plot (Fig. 4E) also demonstrates very close agreement (with a bias of only 0.474 and SD of 0.594).

**Fig. 5:**
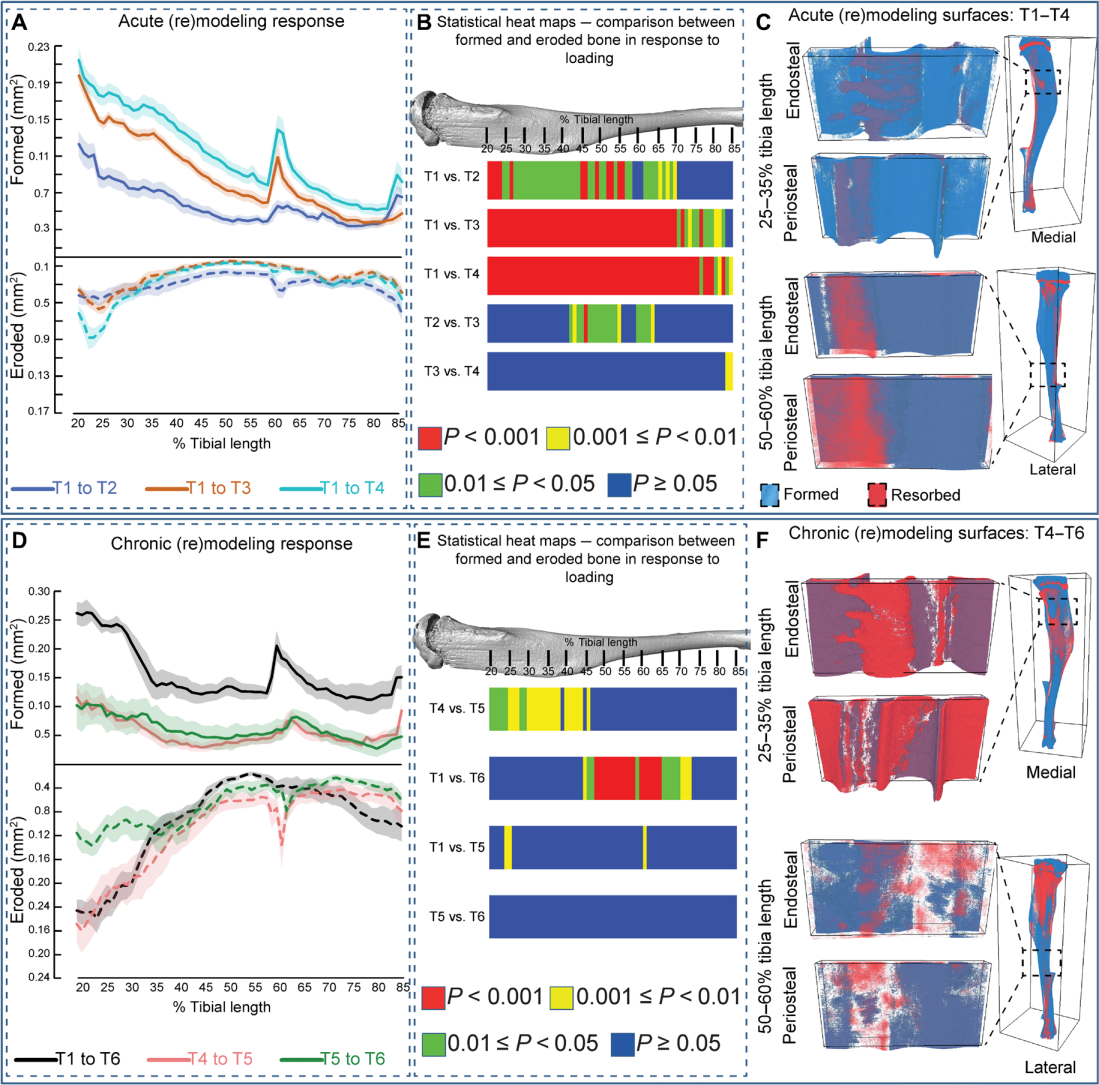
Acute and chronic load-induced bone formation/resorption (re)modeling responses in control and loaded mice. (**A**) Acute kinetic of bone formation and resorption in the control group. (**B**) Statistical heat maps illustrating acute differences between bone formation and resorption. (**C**) Unwrapped color-coded (blue, formation; red, resorption) endosteal and periosteal surfaces of acute load-related (re)modeling responses. (**D**) Chronic kinetic of bone formation and resorption in the control group. (**E**) Statistical heat maps illustrating chronic differences between bone formation and resorption. (**F**) Unwrapped color-coded (blue, formation; red, resorption) endosteal and periosteal surfaces of chronic load-related (re)modeling responses. Red, *P* < 0.001; yellow, 0.001 ≤ *P* < 0.01; green, 0.01 ≤ *P* < 0.05; blue, *P* ≥ 0.05. Line graphs represent means ± SEM.

Using this 3D registration method, we find that the earliest net load-induced bone gains (T1–T2) primarily involve higher formation and lower resorption (~20 to 70%; Fig. 5, A and B), while later acute-phase (T3–T4) minor bone gains at ~85% of tibia length are due primarily to the higher formation. Loading, thus, acutely modifies remodeling to raise formation and lower resorption along much of loaded tibia length. Representative “unwrapped” periosteal/endosteal surfaces at 25 to 35% and 50 to 60% demonstrate this acute effect of loading similarly in both regions (Fig. 5C).

3D registration–based analysis of the chronic phase reveals greater resorption in proximal tibia regions within 6 weeks after load (T4–T5, ~20 to 45%; Fig. 5, D and E), which represents a reversal of the bone gains accrued in the acute phase. Evaluation of formation and resorption in this proximal region between 6 and 12 weeks after load (T5–T6) demonstrates relative quiescence. In contrast, net load-induced bone gains at ~45 to 75% of length arise because of a combination of higher formation and lower resorption (T1–T6). Analysis of periosteal/endosteal surfaces during the chronic postload period (T4–T6) demonstrates higher resorption than formation at 25 to 35% than is evident at 50 to 60% (Fig. 5C), showing that load-induced bone gains in the proximal tibia are reversed, whereas those accrued in midshaft/distal regions are retained during the 12-week-long chronic phase. Our data indicate that loading yields rapid (acute) net gains in the proximal tibia, involving proformative and antiresorptive activities that are both rapidly reversed by the chronic removal of this load stimulus. In contrast, lasting net load-induced gains in midshaft/distal tibia regions involve predominantly an enhanced formation, with only minor subsequent increases in resorption in the chronic postload phase.

### Load-related remodeling is coordinated at the organ level

**Fig. 6:**
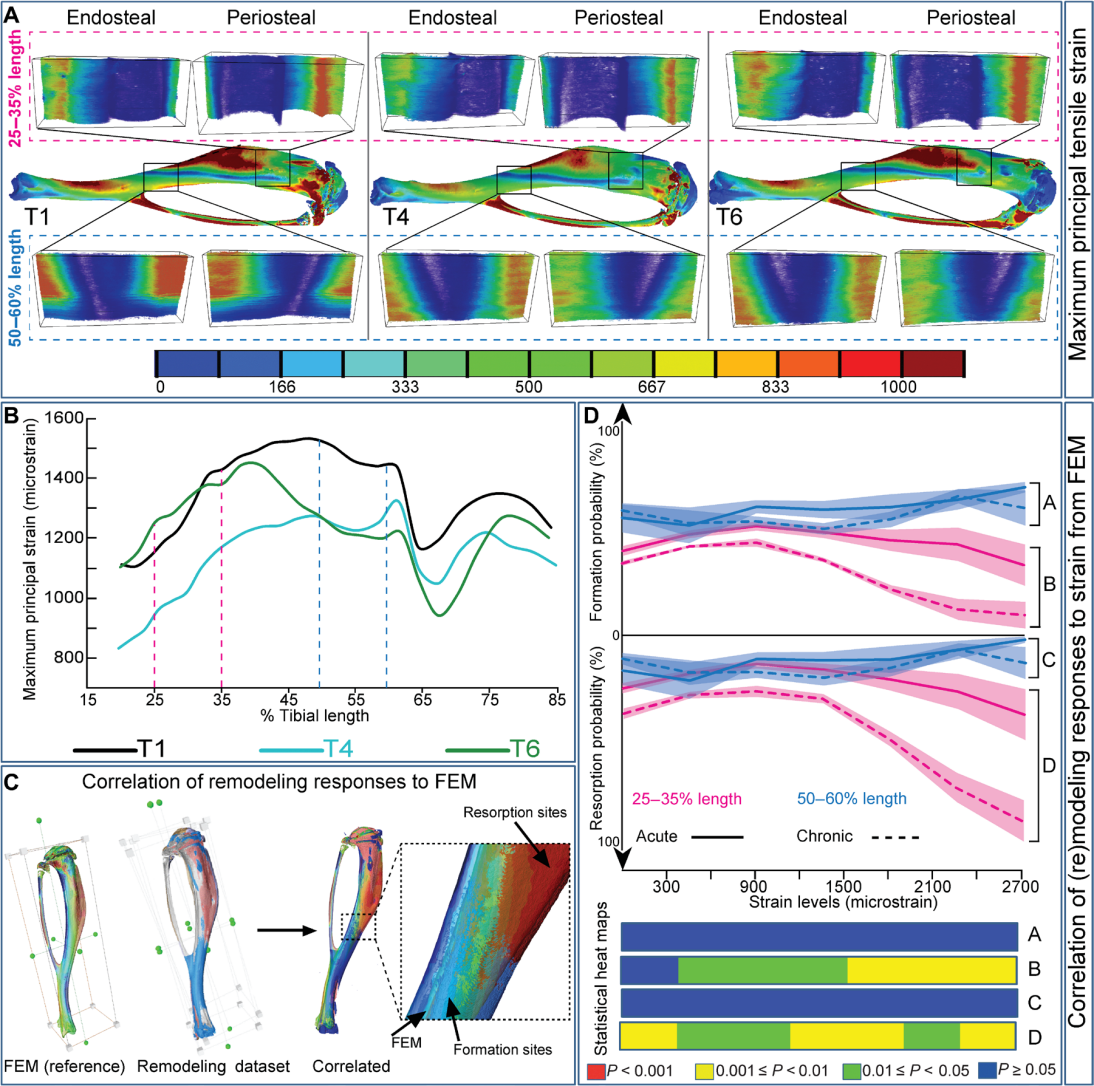
Load-induced modification in tibial architecture is integrated at the organ level. (**A**) Maximum principal tensile strain in unwrapped periosteal and endosteal surfaces of the loaded tibia at 25 to 35% and 50 to 60% of length at T1, T4, and T6. (**B**) The maximum principal tensile strain between 20 and 85% of tibial length at T1, T4, and T6. (**C**) Image processing methodology for correlation of the mechanical strain to the (re)modeling events. (**D**) Probability of acute (solid) and chronic (dashed) bone formation (top) and resorption (bottom) at 25 to 35% (magenta) and 50 to 60% (blue) of tibial length at each available tensile strain (maximum principal strain). Statistical heat maps demonstrate differences between acute and chronic formations at 50 to 60% and 25 to 35% length [(A) and (B), respectively] and acute and chronic resorptions at 50 to 60% and 25 to 35% length [(C) and (D), respectively]. Red, *P* < 0.001; yellow, 0.001 ≤ *P* < 0.01; green, 0.01 ≤ *P* < 0.05; blue, *P* ≥ 0.05. Line graphs represent means ± SEM.

To explore whether relationships with mechanical strain underpin load-induced changes in architecture, we used FEM. Strain mapping revealed that by the end of the acute phase, loading generated architectures that limit local strains along almost the entire tibia (T1–T4, 20 to 85%; except close to the tibiofibular junction at ~65%; Fig. 6, A and B). FEM at the end of the chronic phase (T6) revealed that these lower strains (at T4) reverted to original, T1 levels at the proximal tibia (~20 to 35%), but that no such reversal was observed at the midshaft (50 to 60%). This indicates that the low strain “benefits” accrued acutely were retained chronically in selected regions long after the load stimulus.

To examine the relationship between these FEM strains and local bone remodeling, we measured the number of surface points undergoing formation or resorption, normalized against the total points matching to a specific strain magnitude on the bone surface (quantitative probability, percentage; Fig. 6C). This was assessed in two regions, where load-induced responses were either lost or retained in the chronic phase (25 to 35% and 50 to 60%, respectively; Fig. 6D). In keeping with a reduced probability of formation and conversely greater probability of resorption at higher strains, our data indicate that a linear strain sensitivity underpins the load-induced (re)modeling at 25 to 35% during both the acute and chronic phases. At sites where loading exerts lasting effects (50 to 60%), we find formation probability is higher than resorption and both are unchanged and independent of strain magnitude across acute and chronic phases, indicating that (re)modeling events and strain magnitude are unrelated at this location of the tibia (Fig. 6D). Thus, these strain-(re)modeling relationships demonstrate that stable, but not reversible, mechanoadaptive changes in bone architecture are nonlinear and unrelated to the local strain environment but instead operate at the whole-bone level.

## DISCUSSION

The notion that a bone’s architecture is governed by interactions with mechanical cues, according to strict engineering principles, is described by Wolff’s law (*2*). This is at odds with the curved overall shape of most bones, however, because adherence to these principles would predict straighter bones. This bone design paradox was theoretically deconstructed by Bertram and Biewener (*3*), who hypothesized that the shape of bones is instead optimized for load predictability. This would lead to the prediction that as bone curvature increases and predictability of the bending direction is augmented, its strength decreases (*3*). Despite previous attempts, experimental strategies have yet to establish the organ-level bone shape changes that are engendered by a controlled applied mechanical load over time. We report that bone acts as an organ to acquire lasting modifications in shape and strength with greater bending predictability, which involves coordination of spatial remodeling that is unrelated to local strain magnitude.

The whole-bone shape has been considered the product of temporally phased remodeling events, best described by a mechanostat strain–controlled feedback theory proposed by Frost (*7*). Many studies investigating this proposal have been limited to an examination of only small segments of a whole skeletal element and therefore provide a relatively restricted view of how the remodeling events are coordinated to control bone shape at the organ level. Moreover, the temporal phasing and 3D spatial distribution of these dynamic remodeling events have, due to the need for either multiple large-animal cohorts or the impracticality of longitudinal studies, also remained undefined.

An improved and validated new approach to measuring the dynamics and distribution of bone formation and resorption is to monitor changes by 3D registration of serial images obtained longitudinally using in vivo microCT (*19*). This approach has never been applied, however, at the bone organ level, and thus, it remains to be established whether the mechanostat concept applies equally at all bone scales. We have, therefore, taken a nonbiased, whole-bone approach to directly monitor the relationship between loading and acute and chronic, organ-level bone shape changes to address the following questions: (i) Does loading engender chronic changes in bone structure or in accord with mechanostat theory, and are all the benefits of mechanoadaptation lost following cessation of load? (ii) Which remodeling processes dominate these short- and long-term load-induced adaptive phases? and (iii) What is the relationship between local strain magnitude and these remodeling events?

Since longitudinal monitoring of load-induced bone changes is rarely undertaken, most previous studies including our own have, almost arbitrarily, selected specific short-term duration for examination (*20*, *21*). Early studies had found that a transient, saturable stimulus was sufficient to induce antiresorptive/osteogenic responses (*22*). This was consistent with the very rapid activation of osteocytes that was considered responsible for mechanotransduction (*23*) and with somewhat later, nonetheless acute, emergence of the “functional” osteogenic sequelae (*24*); chronic responses have remained relatively underexplored. To ensure that our studies would encompass both short- and long-term bone responses to load, we have herein chosen to demarcate the longitudinal evaluation of experimental mechanoadaptation into an acute phase, based on the most frequently used time frame, and a chronic phase that extended to double the animal’s age at experimental onset.

Growth-related evaluation of control tibiae in the acute phase disclosed no significant modification in shape and only small increases in mass that were entirely reversed at advanced ages (chronic). While loaded tibiae acutely exhibit amplification of these changes in bone mass attributable to growth, they additionally acquire a lasting organ-level shape modification through the chronic phase, with greater proximal curvature coupled to a straighter design distal to the tibiofibular junction. The rapid and highly significant increases in proximal bone mass observed in the first week are an extension to the many previous studies examining load-related changes after 2 weeks (*12*, *14*, *20*, *21*). Further acute increases in bone mass are only accrued at the mid-to-distal tibia in the postload period (T3–T4). Our tracking of the longevity of these responses reveals two new aspects of mechanoadaptation: (i) that the one accrued initially in response to loading is lost most rapidly during chronic periods (proximally) and (ii) that this chronic phase reversal is not always evident (mid-to-distal). Our data are at odds with the current understanding of mechanostat theory and provide the first experimental support for the hypothesis that the shape of bones is instead optimized for load predictability without sacrificing strength.

Some earlier elegant 2D studies, restricted to small bone segments, described lasting effects of mechanical loading on bone architecture (*25*, *26*). Honda *et al.* (*25*) found that 8 weeks of jump training led to increased bone mineral content/density that was still partially preserved 24 weeks after return to “sedentary” cage activity. Warden *et al.* (*27*) used 2D peripheral quantitative CT analysis to report raised *I*_min_ in distal but not proximal loaded ulnar regions of young growing mice. In addition, a cross-sectional study at only the tibia midshaft showed long-term expansion of the medullary cavity without lasting effects of load on cortical bone mass, which were lost by 26 weeks of detraining (*26*). While these data similarly questioned aspects of the mechanostat theory, they failed to incorporate 3D tracking within an individual bone in a longitudinal controlled setting, which has allowed us to provide experimental evidence to deconstruct the bone design paradox and for lasting changes in long bone curvature in response to a transient loading stimulus.

To establish whether specific targeting of bone resorption or formation differentially distinguishes the reversible from the lasting effects of loading, we used an extensively validated 4D assessment based on spatial registration of a longitudinal series of microCT images (*18*). This assessment revealed that applied loads led to the enhanced formation and reduced resorption around the proximal bone region during the acute phase, indicating that rapid, regionally matched bone modeling underpins the acute mechanoadaptive response. This suggests that local signals drive greater osteoblast and reduced osteoclast activities and is aligned with reports that formation and resorption are uncoupled in these bone modeling processes (*28*). That the area formed is greater than the area resorbed in this acute phase is consistent with a net boost in bone mass. In close agreement with conclusions from previous reports (*29*, *30*), we find that acute bone gains are lost within 6 weeks after load and extend this by showing that this is achieved through a simple reversal, involving greater resorption and lower formation in the proximal tibia in the chronic phase. This, along with the lack of any further changes in formation or resorption from 6 to 12 weeks after load, is evidence that this proximal tibia architecture is restabilized in accordance with predictions based on the mechanostat.

The net load-induced acute gains in the mid-to-distal tibia are, in contrast, lasting. Our 4D analyses reveal that this gain in bone mass is achieved predominantly by an enhanced formation in the acute phase, with only minor subsequent modifications in remodeling in the chronic postload phase. Comparing the remodeling activities in these two diverse, proximal and distal, locations by our novel application of whole-bone 4D analyses leads us to propose that the rapidity of load-induced acute changes in bone remodeling and the rate of their chronic postload reversal are linked. Reversibility in the proximal tibia is aligned to a marked load-related antiresorptive effect, while, contrarily, the lasting adaptation in the mid-to-distal region is principally linked with load-induced osteogenesis. It is intriguing that only the latter leads to permanent modification in bone shape.

Are the (re)modeling events linked to these reversible and lasting bone changes both guided by local strains? Our analyses show that both resorption and formation events exhibit a linear strain sensitivity during the reversible load-related changes in the proximal region. Linear relationships with strain magnitude are consistent with previous work restricted to this acute phase. Thus, Razi *et al.* (*9*) and Schulte *et al.* (*11*) both used longitudinal CT analysis of small bone segments, at the tibial mid-diaphyseal and vertebral trabecular bone, respectively, to find that bone formation most likely occurs at sites of high strain, and resorption at sites of low local mechanical strain. We report that, in contrast, there is a nonlinear independence of local strain magnitude for the mid-to-distal responses, suggesting that the regulation of bone formation and resorption underpinning these lasting architectural changes operates instead at an alternative scale. We propose based on the measured changes in tibial curvature that these lasting remodeling responses function instead to regulate whole-bone load predictability, independently of local strain to better equip the bone to a range of axial and shear stress (*3*). The accompanying increases in bone strength that we observe in these locations are, in addition, no longer adherent to the principles defined by the mechanostat.

By what mechanism can the chronic response be nonlinear? The lasting changes in tibial curvature that we describe herein are indeed in keeping with several historical viewpoints that have never before been tested experimentally. Lanyon and Rubin (*22*) suggested that curvature determines the deformation and that axial loading of straight bones increases the likelihood of disadvantageous buckling. Currey (*31*) also proposed that curvature may better predict approaching strain limits despite the sacrifice in absolute load-bearing strength. Lanyon (*32*) had previously proposed that curvature may be a way in which customary strain values, although greater, could be brought within a particular range. This would effectively protect against aberrant loading that would otherwise jeopardize bones optimized only for axial load by bestowing the structural advantage, whereby they could instead attenuate and absorb rather than transmit skeletal forces (*32*). Another theory to account for curvature proposes that bones are instead optimized for load predictability (*3*). This describes how centric, axial compressive load produces even stress distribution, where all portions of a bone cross section carry an equal proportion of the stress. A straight bone exposed to load of random direction, relative to the bone axis, will also be subject to bending moments equally in all directions, and no restriction on the distribution of stresses will, thus, exist. Curved bones, in contrast, will almost always bend in the direction of the curvature regardless of any variability in the direction of eccentric loading, resulting in a highly predictable strain pattern. Our data are consistent with a load-related emergence of load predictability to avoid failure.

Long-term curvature changes in nonbiological materials can involve creep deformation. Does this provide an alternative explanation for the changes in curvature that we observe? On the basis of our experimental framework and considering the load duration, magnitude, and damage that might accrue in response to load, this explanation appears highly unlikely. While creep tests in cortical bone require many minutes of continuous load, our protocol applies only 12 s of loading over a 2-week-long period. Further, secondary creep failure behavior of bone starts at ~1% strain (*33*), but, in contrast, strain gauging and FEM show that maximum strains induced in the tibia in our setup reach ~0.2% strain, well below any creep damage threshold. Lack of load-induced intracortical fluorophore labeling, a proxy measure of microdamage, and an absence of basic fuchsin microcrack staining in the loading model we have used (*15*) are inconsistent with creep. The timing of the structural changes we have found is also inconsistent with creep, because there are no significant changes in overall curvature between T1 and T3 (day 14), when the tibia has already been loaded for six episodes. Tibial curvature is only significantly greater than at day 14 (T3), 12 weeks (T6) after load cessation. While submicroscopic creep damage is possible, our data do not fit with the idea that this underpins the long-term curvature changes.

Thus, by whatever mechanism the bone is exhibiting this nonlinear chronic response to strain, the resultant tibial morphology is evolutionarily selected to have its characteristic curved shape, conferring the advantage of predictability. Nonlinearity is a known phenomenon in growth and morphogenesis that can also operate in the bone (*34*). One feature of nonlinear pattern formation is convergence to “attractors.” If these behaviors were being exhibited by the bone in its chronic response to load, then the mouse tibia would not be free to change to any shape. Only a subset of shapes is possible—these are the attractors. There is no attractor for a straight cylinder, for example. Thus, loading appears to adaptively exaggerate the bone’s inherent shape to provide greater protection against habitual loading exposure; curved tibial regions proximal to the tibiofibular junction become curvier, and the straighter distal regions even more upright. One very strong attractor in tibial bone design is indeed the tibiofibular junction. It has been shown that tibial growth isometrically scales to maintain the position of this junction (*35*). We speculate that there is a programmed shape for a bone and that the adaptation to load exhibits only limited freedom to depart from this shape. The sites that diverge most readily from the predictions made by the mechanostat are those that serve to maintain these attractor functions. Hence, the lasting changes in load-induced bone shape, as well as underpinning (re)modeling, fail to adhere to strain linearity and accumulate around the tibiofibular junction.

Our findings may provide new insights with clinical impact. Thus, it is possible that the monitoring of rapid bone modifications induced in response to skeletal surgical procedures may not be truly indicative of the potential long-term benefits that might be accrued and suggests that follow-up tracking of bone shape modifications may be meaningful. Our findings may be relevant when monitoring the beneficial effects of exercise on skeletal health, which are often restricted to the monitoring of highly localized bone mass changes at specific locations. Our data suggest that overall 3D bone shape monitoring may be a greater predictor of structural benefit. Likewise, osteoporosis trials often focus on common fracture sites, rarely respecting a potential mechanical etiology of the enhanced risk. It is possible that the failure to retain appropriate curvature levels dictates this fracture risk and that load-related bone shaping is what becomes deficient in these patients. Further, existing osteoporosis treatments do not currently target the bone regions that most markedly contribute to bending strength; they instead have generalized antiresorptive/proformative effects. Recent interventional loading studies in aged animals (*36*) and a randomized, controlled, 20-week-long high-intensity strength/sprint training trial in older male sprint athletes, which found significant improvements in midtibial (not distal) structure/strength (*37*), suggest that the effects of loading can indeed be targeted to limit the age-related decline in bone strength. This implies that new, more “intelligent” bone therapies that interact with mechanical load to more selectively preserve overall curvature to resist fracture are potentially identifiable.

This is the first report that examines the long-term, longitudinal structural adaptation of a whole bone over both acute and chronic phases to explore the paradox in bone curvature. We show that some acute load-induced structural changes are reversible and adhere to linear strain magnitude feedback-loop regulation of remodeling activities. On the other hand, a vast proportion of the bone retains lasting structural memory of loading to generate nonlinear, strain magnitude–independent remodeling to achieve greater curvature optimized for load predictability without sacrificing strength.

## MATERIALS AND METHODS

### Animals

Female C57BL/J6 mice (Charles River, UK) were housed in polypropylene cages under 12-hour light/dark cycle at 21° ± 2°C with free access to rat/mouse 1 maintenance diet (Special Diet Services, Witham, UK) and water ad libitum. Mice were randomly separated into two groups: 18 mice in the loading group and 6 mice in the control group. All procedures were carried out in accordance with the Animals (Scientific Procedures) Act 1986, an Act of Parliament of the United Kingdom, approved by the Royal Veterinary College Ethical Review Committee and the United Kingdom Government Home Office under specific project license, and followed ARRIVE (Animal Research: Reporting of In Vivo Experiments) guidelines.

### In vivo loading

Apparatus and protocol for dynamic tibial loading were described previously (*15*). Briefly, under isoflurane-induced anesthesia, the right tibia of each of 18 female mice at 12 weeks of age was held in the cups, and dynamic (12 N, 40 cycles/day, 2 Hz, with 10 s rest periods between cycles) axial loads were applied through the knee on alternate days for 2 weeks—six loading episodes in total. The nonloaded right tibiae of six mice served as control. This loading magnitude is well below the critical buckling stress and fracture point as demonstrated in fig. S3. The loaded mice were able to move around in the cage and gained access to food and water without difficulties, and no adverse effect was observed. To assess histomorphometry, mice received an intraperitoneal injection of alizarin red (20 mg/kg; Sigma-Aldrich, MO, USA) 10 and 3 days before euthanasia and collection of bone samples.

### In vivo monitoring of bone remodeling using microCT

In vivo scanning of the entire right tibia was achieved using SkyScan 1176 (SkyScan, Kontich, Belgium). The x-ray tube operated at 40 kV, 600 μA, with 1-mm aluminum filter, voxel size of 9 μm, orbit of 198°, and a rotation step of 0.80°. At 12 weeks of age and 3 days before the start of loading (day 0: T1), 18 mice in the loading group underwent in vivo scanning under isoflurane-induced anesthesia. The scans were repeated on T2, T3, and T4 (days 7, 14, and 17 respectively). Six weeks after the end of loading episode (day 59: T5), mice were scanned. The final scan was performed 12 weeks after loading (T6). Mice in the control group (*n* = 6) were subjected to the same scanning regime. The radiation dose from the microCT scanning was estimated to be approximately 500 mGy for each scan, which has been proved to cause no significant effect on bone adaptations (*38*).

### 3D image registration

To quantify mechanoadaptive and growth-related remodeling events, microCT datasets obtained from different time points (T1–T6) were registered using DataViewer. Initially and after the acquisition, cone beam reconstruction based on the Feldkamp algorithm was performed using NRecon (SkyScan, Kontich, Belgium). Before registration and regardless of the position of bone in the scanner, microCT images were geometrically aligned in common coordinates such that the cross section within the transverse plane was perpendicular to the long axis of the bone. Multiple 3D rigid registrations of “reference” and “target” datasets were performed (nine combinations: T1–T2, T1–T3, T1–T4, T1–T5, T1–T6, T2–T3, T3–T4, T4–T5, and T5–T6) using DataViewer with shift range at 50, rotation range in 2D at 45 and 3 iterations of alignment. Registered referenced and target images were exported.

### 3D microCT-based dynamic morphometry

To measure dynamic 3D remodeling between consecutive datasets, exported registered images were binarized, manually segmented, and subjected to bitwise operations to extract formed (bone absent in reference but present in target) and eroded surfaces (bone present in reference but absent in target scans); areas of the bone present only in earlier scans are considered resorbed bone, while those only present in later scans corresponded to newly formed bone (*17*). These operations were performed along the entire length of the tibia, and the resulting formed and eroded surfaces were quantified using 2D slide-by-slide analyses (CTAn 1.15+, SkyScan, Kontich, Belgium). The first 20% proximal and the last 10% distal of the tibia were excluded from the analysis to avoid inclusion of the trabecular bone in the analysis of cortex.

### 3D microCT-based static morphometry

The whole-bone analysis was performed using BoneJ (version 1.4.0, an ImageJ plugin). Following segmentation, alignment, and fibula removal from the dataset, a minimum threshold was used in “Slice Geometry” (BoneJ) to calculate measures of mass: CSA, mean thickness (Ct.Th), and shape, namely, second moment of area around the major (*I*_min_) and minor axes (*I*_max_), ellipticity, and predicted resistance to torsion (*J*).

### Validation of image processing methodology

To validate the accuracy of data obtained from 3D registration, two steps were taken.

#### Validation with 2D dynamic histomorphometry

Loaded tibiae (*n* = 4) were dissected, fixed in 4% formaldehyde (Alfa Aesar Inc., Ward Hill, MA, USA), and stored in 70% EtOH. Before embedding, tibiae were washed, dehydrated through graded concentrations of alcohol, infiltrated, methyl methacrylate embedded, and sectioned at a thickness of 7 μm. Fluorochrome labels were observed using a Leica Q550IW fluorescence microscope with a DC 500 Leica digital camera. The distance between the inner and outer fluorescent labels at increments of approximately 5-μm part was measured in multiple sections per mouse and averaged. Comparable images from microCT-based morphometry and histomorphometry were visualized. MAR was calculated by dividing this distance by the labeling period (7 days). In addition, the MAR calculated from 3D microCT-based morphometry was quantified by measuring the average and maximum thickness of formed surfaces and plotted against histomorphometrically based MAR, and their differences were visualized using the Bland-Altman plot.

#### Validation with repeat scan and repeat registration of same datasets

To confirm variability and reproducibility of registration methodology, 3D registrations were performed on datasets obtained from repeat scans and on repeat registration of the same datasets. Briefly, scanning of mice was repeated, and datasets obtained were rigidly registered as described. In addition, rigid registration of the same scans was performed; both reference and target datasets were the same.

### Determining bone curvature

An in-house script written in MATLAB 2015a (The MathWorks Inc., Natick, MA, USA) was used to determine the bone curvature. The script calculates the centroid of bone at each cross section and determines its minimum Euclidian distance to the bone longitudinal axis. The tibial longitudinal axis (proximal-distal axis) was defined as the axis passing through the midpoints between the medial and lateral eminences of the upper tibia (on the sides of tibial tuberosity) and the medial and lateral malleolus.

### Relating (re)modeling events with corresponding local mechanical strains

#### Finite element models

Local mechanical strains induced in the loading experiment within the loaded mouse tibiae were determined using FE analyses (Abaqus 6.14-5, Dassault Systèmes, Providence, RI, USA) as described previously (*39*, *40*). Briefly, representative entire tibia microCT data from each time points were selected to build FE models corresponding to the T1, T2, T3, T4, T5, and T6 time points in loaded limbs. Boundary conditions were set to replicate the experimental setup (*15*). The mechanical environment induced within the experimentally loaded limbs was determined by the application of −12-N force at the boundary regions (i.e., knee contact surfaces). Distal boundary regions (i.e., ankle contact surfaces) were constrained from movement. Tibial elastic properties were considered to be isotropic but inhomogeneous along tibial length. Bone tissue mineral density is known to be a relevant predictor of elastic modulus in bone. We considered the variations in the tissue mineral density (i.e., changes in the local linear attenuation coefficients captured by microCT) along the tibia length as estimators of regional differences in local elastic moduli (*39*). Relative regional elastic modulus along the tibia length (i.e., homogeneous in each cross section of bone) was calculated using

ERaERb=(μ^aμ^b)1.5

in which *E_Ra_* and *E_Rb_* are the average elastic modulus in regions of interest *R_a_* and *R_b_*, respectively, and ${\hat{\mu }}_{a}$ and ${\hat{\mu }}_{a}$ are average linear attenuation coefficients in *R_a_* and *R_b_*, respectively (*39*). As a result, 21 regions of homogeneous material properties, including fibula, were assigned within the models. Young’s modulus assigned in different regions of tibia ranges from 3.6 GPa (i.e., the low-density region at the proximal tibia) to 16.72 GPa (at the midshaft). This methodology is exhaustively described in Razi *et al.* (*39*).

#### Nearest neighboring strains to formation or resorption

(Re)modeling events determined at the T4–T1, T6–T1, and T6–T4 time points (*n* = 6 per time point) were mapped onto the FE models of the T1 and T4 time points to match changes both during the acute and chronic phases to the original T1 time point and those changes occurring in the latter chronic phase to those already accrued during the acute phase. To identify the nearest mechanical stimulation corresponding to each of the (re)modeling events, an established numerical methodology was used (*9*). Briefly, the voxel-based data were mapped and transformed to the tetrahedral grid of the FE models to register (re)modeling events onto FE models. Initially, the entire outer surface of the (re)modeling events and the FE models were extracted to perform a stepwise affine registration approach. This approach interpolates the (re)modeling labels into the same coordinates as the FE model grid (Fig. 6C). Computed strain values at each (re)modeling site were determined by finding the closest integration node in the FE model grid to each (re)modeling event (representing a single voxel classified as formed, resorbed, or quiescent). This was performed using the nearest neighborhood algorithm written in MATLAB 2015a (The MathWorks Inc., Natick, MA, USA). The inclusion criterion for the minima was a distance less than 1 μm. Last, for each pixel at the bone surface representing an occurrence of a (re)modeling event, one point at the surface of the FE model was associated.

#### Data analysis

For each (re)modeling event, maximum (tensile), minimum (compressive), and absolute maximum (i.e., either tensile or compressive with a larger eigenvalue) principal strains were determined. Using the methodology described, the probability of a given (re)modeling event to occur at a specific strain level was calculated by dividing the number of (re)modeling events at a specific strain magnitude to the total number of available spots at the surface under that specific strain magnitude. Surface extraction and registration and pre/postprocessing were performed in ZIBAmira (Zuse Institute, Berlin, Germany) and MATLAB 2015a, respectively.

### Statistical analysis

Programming language “R” 3.3.2. (R Foundation for Statistical Computing, Vienna, Austria) was used to generate graphs on identical scales for comparison. Linear mixed-effects model(s) with fixed effect for time and random mouse effect was performed on values corresponding to every 1% along the length of the tibia; Fisher’s least significant difference post hoc comparison was used. Normality and heterogeneity of the variables were assessed using Bartlett’s and Shapiro-Wilk tests. “R” also generated heat maps for the *P* value at each percentage of the tibia length. Paired *t* test and Bland-Altman plot were used to compare MAR from 2D histology and 3D histomorphometry (GraphPad Prism). Significance level was set at *P* < 0.05 (two-tailed).

## Supplementary Material

aax8301_SM.pdf

## SUPPLEMENTARY MATERIALS

Supplementary material for this article is available at http://advances.sciencemag.org/cgi/content/full/6/10/eaax8301/DC1 Fig. S1. Acute and chronic load-induced changes in tibial ellipticity along the entire tibia length. Fig. S2. Acute and chronic load-induced adaptation of mean cross-sectional thickness along the entire tibia length. Fig. S3. Load-displacement curve to demonstrate how 12-N load magnitude used in our studies relates to bone failure.

## Appendix

View/request a protocol for this paper from *Bio-protocol*.
