# Supplementary material for: Lasting organ-level bone mechanoadaptation is unrelated to local strain
Source: Sci Adv. 2020 Mar 6;6(10):eaax8301. doi: 10.1126/sciadv.aax8301 (PMC7060058; doi:10.1126/sciadv.aax8301)
Supplement: aax8301_SM.pdf [file aax8301_SM.pdf]

Science Advances
AAAS

advances.sciencemag.org/cgi/content/full/6/10/eaax8301/DC1

# Supplementary Materials for

## Lasting organ-level bone mechanoadaptation is unrelated to local strain

Behzad Javaheri, Hajar Razi, Stephanie Gohin, Sebastian Wylie, Yu-Mei Chang, Phil Salmon,
Peter D. Lee, Andrew A. Pitsillides*

*Corresponding author. Email: apitsillides@rvc.ac.uk

Published 6 March 2020, *Sci. Adv.* **6**, eaax8301 (2020)
DOI: 10.1126/sciadv.aax8301

**This PDF file includes:**

Fig. S1. Acute and chronic load-induced changes in tibial ellipticity along the entire tibia length.
Fig. S2. Acute and chronic load-induced adaptation of mean cross-sectional thickness along the entire tibia length.
Fig. S3. Load-displacement curve to demonstrate how 12-N load magnitude used in our studies relates to bone failure.

**SUPPLEMENTARY MATERIALS**

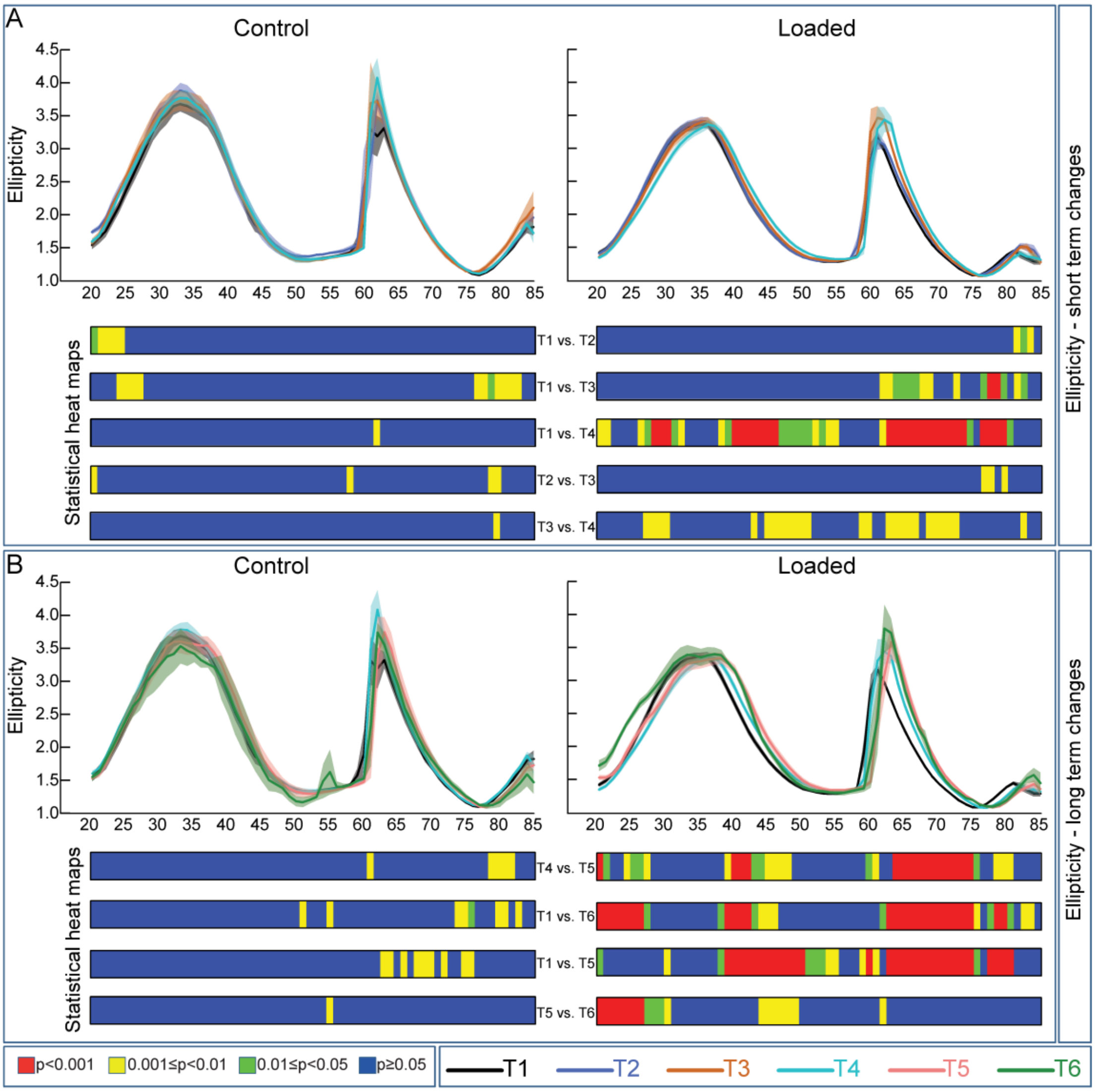


**Fig. S1. Acute and chronic load-induced changes in tibial ellipticity along the entire tibia length.** (**A**) The ellipticity of control and loaded tibiae in female C57/Bl6 at T1-4. (**B**) The ellipticity of both groups at T1, T4-6 demonstrating chronic mechanoadaptation. Statistical significance of differences in ellipticity between different T's within the group along the entire tibia shaft represented as a heat map. Red $p<0.001$, yellow $0.001\leq p<0.01$, green $0.01\leq p<0.05$ and blue $p \geq 0.05$. Line graphs represent means ± SEM.

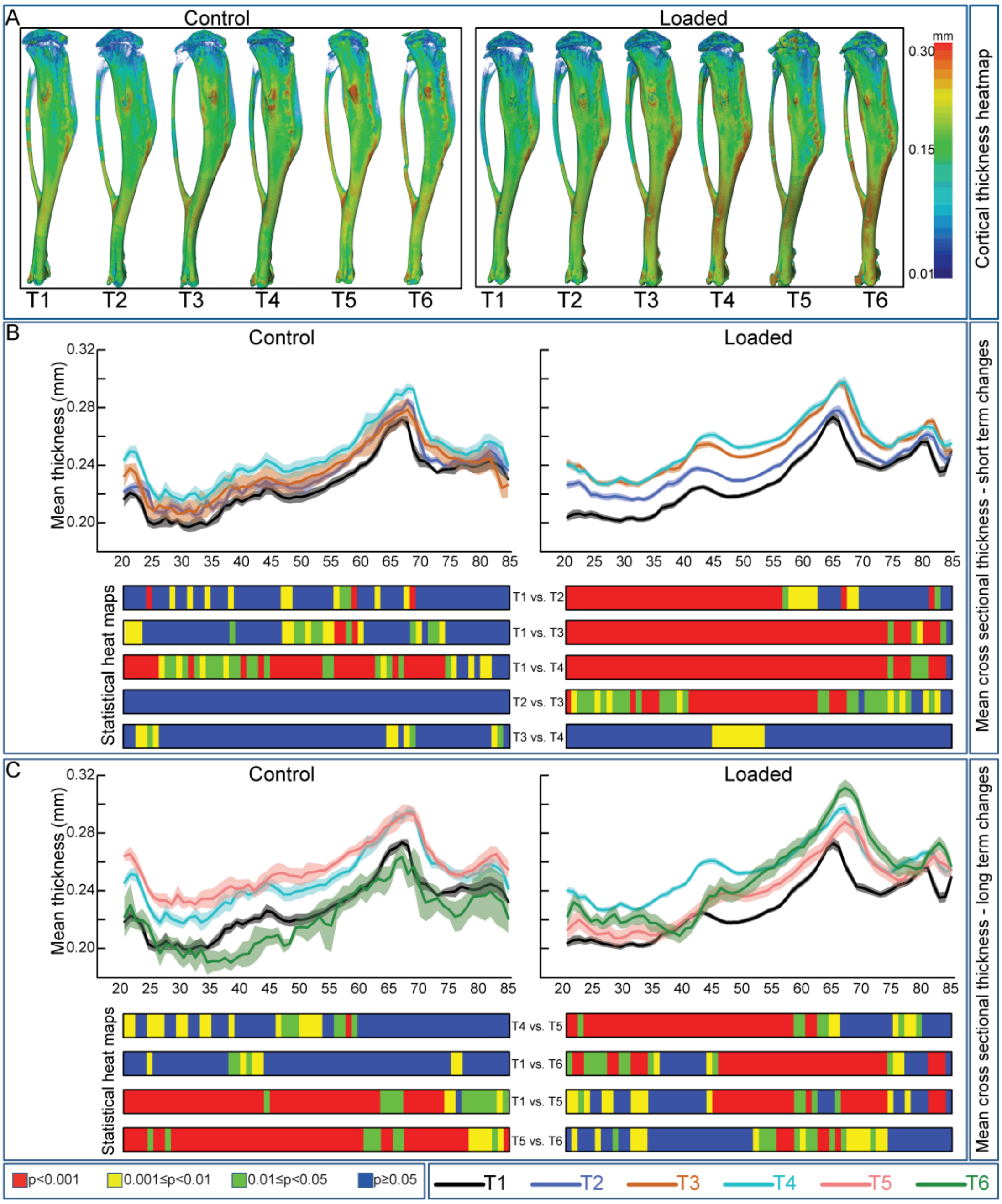


**Fig. S2. Acute and chronic load-induced adaptation of mean cross-sectional thickness along the entire tibia length.** (**A**) Representative 3D micro-CT colour-coded images of tibia cortical bone thickness of both control and loaded mice at T1-6. (**B**) Mean thickness of control and loaded tibiae in female C57/Bl6 at T1-4. (**C**) Mean thickness of both groups at T1, T4-6 demonstrating chronic mechanoadaptation. Statistical significance of differences in thickness between different T's within the group along the entire tibia shaft represented as a heat map. Red $p<0.001$, yellow $0.001 \leq p<0.01$, green $0.01 \leq p<0.05$ and blue $p \geq 0.05$. Line graphs represent means ± SEM.

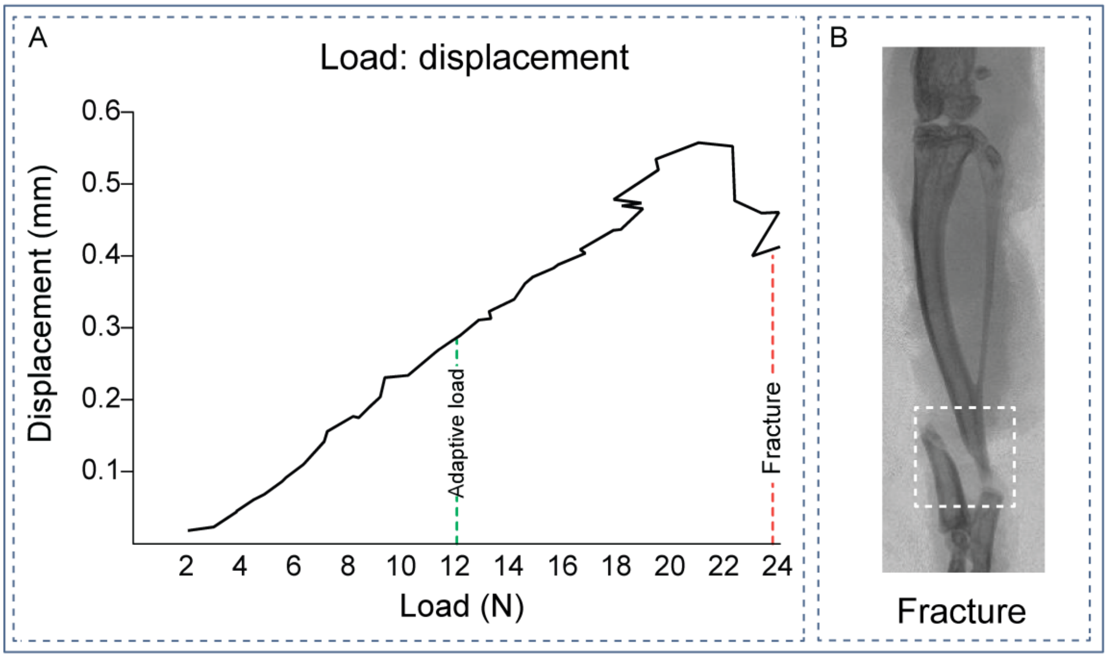


**Fig. S3. Load-displacement curve to demonstrate how 12-N load magnitude used in our studies relates to bone failure.** (**A**) Load: displacement graph demonstrating the relationship between the magnitude of load and tibial displacement. (**B**) MicroCT scout-view of tibia fractured at 24N.
